# Additive Manufacturing of Nerve Guidance Conduits for Regeneration of Injured Peripheral Nerves

**DOI:** 10.3389/fbioe.2020.590596

**Published:** 2020-09-25

**Authors:** Shaochen Song, Xuejie Wang, Tiejun Wang, Qinghua Yu, Zheyu Hou, Zhe Zhu, Rui Li

**Affiliations:** ^1^Department of Hand Surgery, The Second Hospital of Jilin University, Changchun, China; ^2^Department of Orthopaedic Traumatology, The First Hospital of Jilin University, Changchun, China; ^3^Department of Burn Surgery, The First Hospital of Jilin University, Changchun, China

**Keywords:** biomaterial, additive manufacturing, nerve guidance conduit, nerve regeneration, peripheral nerve

## Abstract

As a common and frequent clinical disease, peripheral nerve defect has caused a serious social burden, which is characterized by poor curative effect, long course of treatment and high cost. Nerve autografting is first-line treatment of peripheral nerve injuries (PNIs) but can result in loss of function of the donor site, neuroma formation, and prolonged operative time. Nerve guidance conduit (NGC) serves as the most promising alternative to autologous transplantation, but its production process is complicated and it is difficult to effectively combine growth factors and bioactive substances. In recent years, additive manufacturing of NGCs has effectively solved the above problems due to its simple and efficient manufacturing method, and it can be used as the carrier of bioactive substances. This review examines recent advances in additive manufacture of NGCs for PNIs as well as insight into how these approaches could be improved in future studies.

## Introduction

Peripheral nerve injury (PNI) is usually caused by cross-section, extrusion, or stretching (Petcu et al., 2018). The peripheral nervous system is a network of 43 pairs of motor and sensory nerves that connect the brain and spinal cord (central nervous system) to the entire human body. Epidemiological studies have shown that the incidence of peripheral nerve injury can be as high as 3% of all trauma patients (Zhang et al., 2020a). Connective tissue provides mechanical support for nerve fibers and contains blood vessels, which provide nutritional support for nerve fibers. From the inside to the outside, a peripheral nerve can be divided into three layers: endoneurium, perineurium, and epineurium.

## Types of Nerve Injury

Seddon divided PNI into three grades of injuries. Based on the Seddon classification, Sunderland expanded PNI to five degrees of injuries based on the histological changes of the PNI (Sunderland, 1951; Grinsell and Keating, 2014).

Treatment for PNI is dependent upon the extent of the pathophysiological damage and the integrity of supportive structures of the axons, endoneurium, perineurium, and epineurium of the peripheral nerve, and functional outcome (Schmidt and Leach, 2003; Pinho et al., 2016; Petcu et al., 2018). A Sunderland grade-I PNI denotes transient neurological disease. Grade II is characterized by axonal destruction, but the endoneurium is intact. Hence, PNIs of Sunderland grades I and II can recover without intervention. Grade III is associated with additional loss of the endoneurium, whereas the other supporting structures (e.g., perineurium) remain intact. This scenario results in non-specific and misguided regeneration because the newly regenerated axons can randomly enter any of the distal endoneurial pathways in close vicinity (Tos et al., 2015). Spontaneous recovery in grade-III injuries is possible but variable. Sunderland grade-IV injuries are characterized by damage to the endoneurium and epineurium, and are the most severe pathological entities. In grade-V injuries, all the supporting structures are severely damaged, and significant hemorrhage and inter-gap scarring follows the injury. The opportunity for spontaneous recovery in grade-IV and -V injuries is very low, and these lesions require surgical exploration and repair (Grinsell and Keating, 2014).

## Treatment Strategies for PNIs

Although human peripheral nerves can regenerate after injury, this degree of regeneration is limited, and may not achieve a good effect on nerve function (especially for Sunderland grade-IV and -V injuries). Most PNIs require treatment to promote the recovery of nerve function. Treatment methods for PNIs can be divided into two categories: non-surgical and surgical.

### Non-surgical Treatment Methods

Non-surgical treatment methods have many advantages in the treatment of PNIs of Sunderland grade I–III. However, the effect of non-surgical treatment is often uncertain because identifying the type and severity of injury is challenging.

#### Physical Therapy

Physical therapy (e.g., electrical stimulation, magnetic stimulation, laser phototherapy) is considered to be one of the most widely used and efficacious non-surgical treatment methods (Martinez De Albornoz et al., 2011). Electrical stimulation is one of the most popular and tolerated treatment methods. Low-frequency electrical stimulation can promote the recovery of nerve function, but its optimal frequency of use and duration, and side effects are not well characterized. Improper application may even cause adverse results (Al-Majed et al., 2000; O’gara et al., 2006; Geremia et al., 2007; Haastert-Talini et al., 2011).

Magnetic stimulation and laser phototherapy are also widely applied physical therapies. Magnetic stimulation can promote the recovery of PNIs (Bannaga et al., 2002). Laser phototherapy can accelerate the recovery of nerve function and reverse atrophy of the corresponding muscles (Gigo-Benato et al., 2004; Rochkind et al., 2007a, b; Camara et al., 2011).

#### Pharmacotherapy

According to animal experiments, several drugs have been shown to promote the recovery of peripheral-nerve function after injury. However, only a few drugs have been applied clinically, such as neurotrophin 3, glial cell-derived neurotrophic factor (GDNF), glial growth factor, ciliary neurotrophic factor, and leupeptin (Joung et al., 2010). Some studies have demonstrated that a combination of physical therapy and pharmacological therapy can promote regeneration of nerve function, such as a combination of electrical stimulation and corticosteroids (Sharma et al., 2010).

### Methods of Surgical Treatment

The purpose of surgery is to reconstruct the continuity of the endoneurium, perineurium, and epineurium, thereby supporting the regeneration of nerve axons. Injury severity can be determined intraoperatively, so the treatment effect is more specific than that using non-surgical treatment. The common surgical methods are described below.

#### Neurorrhaphy

Neurorrhaphy is the most basic and commonly used surgical method to suture the proximal and distal ends of the perineurium and/or epineurium together (Li et al., 2014). Neurorrhaphy is suitable for nerves without defects or with small defects, and nerves can be sutured without tension. If there are medium-sized and large nerve gaps, the recovery effect is weak due to excessive tension after suture, so nerve grafting or nerve transfer will be needed (Johnson and Soucacos, 2008).

#### Nerve Transfer

“Nerve transfer” is defined as the repair of a distal injury by use of a proximal “foreign” nerve as the donor (Midha, 2006). After the healthy donor nerve is cut, it is transferred to the more critical receptor muscle to rebuild function. Therefore, it is often used in regeneration of upper-limb function and repair of brachial plexus nerves. However, nerve transfer has not been used widely owing to missing innervation of the donor nerve and co-contraction-related complications.

Tissue engineering has been used widely (Zhang et al., 2019; Cui et al., 2020; Qiu et al., 2020; Zhu et al., 2020), especially for tissue repair. Also, polymer-based nerve catheters have become the most promising alternatives to autologous transplantation. With technological advancements in scaffold preparation (Ding et al., 2019; Feng et al., 2019; Zhang et al., 2019; Zhao et al., 2019) [especially using three-dimensional (3D) printing], repair of extremely long nerve defects is expected.

#### Nerve Grafting

Nerve autografts have been considered the “gold standard” for treatment of medium-sized and large defects in peripheral nerves (Ray and Mackinnon, 2010). Commonly used nerves used for donation are the sural nerve, intercostal nerves, superficial and deep peroneal nerves (Norkus et al., 2005). Compared with other methods, nerve autografts are regarded to be efficacious, but the chance of recovery of nerve function is ∼50%. Given the disadvantages associated with this procedure, such as a lack of donor-area sensation and poor matching between the donor nerve and defective nerve (Grinsell and Keating, 2014; Li et al., 2014), application of nerve autografts is limited and better recovery of nerve function is hard to achieve.

As the most promising alternative to autologous transplantation, nerve conduits are currently available in humans for repair of nerve defects less than 3 cm in length. Nerve allografts can avoid the donor-site morbidity caused by autografts, and can be applied more flexibly clinically. The main drawback of allografts is the associated morbidity of immunomodulatory therapy (systemic immunosuppression is a prerequisite for allografting).

## Design Principles for Additive Manufacturing of Nerve Guidance Conduits

The nerve conduit scaffolds mainly refers to the structure of the conduit connecting the proximal and distal ends of the nerve defect. If the nerve conduit combines with cells and/or growth factors, that is, nerve tissue engineering grafts, the length of the nerve defect and the repair effect can be further improved. NGCs connect the distal and proximal ends of a defective nerve. This procedure is a substitute for nerve transplantation, and avoids the limitations and damage caused by nerve transfer and nerve grafting. NGCs not only provide structural support for axon regeneration, they also offer various nerve factors and other regenerative-environment support, thereby promoting nerve regeneration (Mackinnon et al., 1984; Apel et al., 2010). The “ideal” NGC should not only have biomimetic structures to provide structural support for axon growth, but also provide nutritive support at all stages of nerve regeneration while having conductivity, biocompatibility, and degradability. One of the main advantages of 3D-printed NGCs is the ability to “customize” any desired shape and to add suitable active cells. Additive manufacturing of NGCs involves consideration of various factors, as discussed below.

### Biocompatibility and Degradation of Nerve Guidance Conduits Materials

The materials employed for 3D printing of NGCs include biological materials and/or cells. Biomaterials can be from nature or can be synthesized using polymers, ceramics, metals, or composite materials. Several biological materials have been used in the 3D printing of NGCs (Zhuang et al., 2018). However, only a small number of biological materials, such as alginate, chitosan, agarose, a biodegradable polyurethane (PU)-modified poly(ε-caprolactone) (PCL) hydrogel, have been used for the 3D printing of active tissues (Lin et al., 2016; Gu et al., 2017; Zhuang et al., 2018). All materials must non-toxic to cells and tissues, and not elicit inflammatory or immune responses (Wang and Sakiyama-Elbert, 2019).

The materials used for 3D printing of NGCs should have a suitable degradation rate. The ideal NGCs should retain their shape, wait for the axon to grow from the proximal stump through the defect and re-innervate the distal nerve pathway, and then begin to degrade gradually and minimize the pressure on surrounding tissues. If the degradation rate is too fast, it can cause local inflammation. If the degradation rate is too slow, the NGC compresses the nerve, leading to chronic immune rejection. For example, non-degradable materials such as silicone and polytetrafluoroethylene require a second procedure to remove the stent, and fibrotic scars may appear after long-term implantation, which limits the application and widespread use of these materials (Mann and Helbing, 2017).

### Mechanical Properties of Nerve Guidance Conduits

3D-printed NGCs provide mechanical support. NGCs serve as channels for the infiltration of cells and axons, and axon diffusion, in human tissues (Yoshii et al., 2003; Nectow et al., 2012; Dixon et al., 2018; Koffler et al., 2019). Therefore, the mechanical properties of NGCs should be similar to those of peripheral-nerve tissue and surrounding tissues to avoid mechanical damage to these tissues after NGC transplantation. NGCs should not only have a certain degree of anti-compression protection, but also have a certain degree of flexibility to resist the pulling and twisting forces generated during limb activities, thereby protecting the new axons (Chang et al., 2018).

### Microstructure of Nerve Guidance Conduits

The microstructure of NGCs not only affects mechanical properties but also affects the arrangement of cells and axons, as well as the exchange of materials inside and outside the ducts, which is essential to nerve regeneration. Intraluminal microchannels and the permeability of the tube wall are the most popular designs of NGCs (Hanani, 2005).

The microchannels in NGCs are not only channels that support axon growth, but also essential factors affecting the morphology and function of axons (Nectow et al., 2012). Therefore, the microchannels in NGCs need to be large enough to support the growth of axons and blood vessels. Although a microchannel with a large diameter is beneficial to the growth of blood vessels and nerves, it reduces the migration of axons and Schwann cells in NGCs, and factors such as scar ingrowth are not conducive to axon growth. Krych et al. (2009) observed a significant reduction in the number of nerve axons in tubes of diameter > 450 μm, but axon regeneration was observed in microchannel scaffolds with a diameter of 150–300 μm. Several other studies have also shown that axons, blood vessels, and glial cells regenerated in microchannels within a diameter of 100–300 μm can be arranged linearly to achieve effective regeneration of nerves (Stokols et al., 2006; Pawelec et al., 2018; Koffler et al., 2019). As the diameter of the microchannel decreases, the regenerated axons and blood cells can be arranged linearly more effectively. However, a too-small diameter prevents the ingrowth of blood vessels and effective nutrient fluid exchange in NGCs, which is not conducive to nerve regeneration. It is considered that a microchannel diameter of 20–30 μm is appropriate (Deumens et al., 2010; Pawar et al., 2011; Sarker et al., 2018), but more experimental results are needed.

An appropriate permeability of the tube wall should promote the transportation of nutrients and blood supply, isolate the invasion of scar tissue-forming cells, and help discharge metabolic waste (Chiono and Tonda-Turo, 2015; Sarker et al., 2018). Some researchers believe that for repair of peripheral nerves, a micropore size of 10–40 μm and porosity of 80% is the most suitable ratio (Kokai et al., 2009). In addition to pore size, permeability is also affected by the hydrophilicity of the material and the distance from the tube wall to the center.

The surface structure and properties of the material also affect the growth and differentiation of cells. For example, a larger surface roughness (85–200 nm) supports generation of longer axons and more neurite outgrowths/branches than that of a smoother surface (surface roughness of 6–50 nm). For human endothelial cells, a higher surface roughness (35 nm) of biomaterials can enhance the adhesion and growth of cells compared with that obtained with a roughness of 20 nm (Chung et al., 2003). In addition, more hydrophilic surfaces exhibit a higher rate of cell adhesion and tend to absorb more protein. As the surface hydrophobicity decreases, the rate of neuron diffusion and neurite outgrowth increases (Popovich, 2012). Therefore, careful design of the local microenvironment is very important for nerve tissue-engineered scaffolds.

In short, on the basis of satisfying the requirements of biocompatibility, permeability and mechanical properties, the additive manufacturing nerve conduit could be better simulated with extracellular matrix by means of improving the printing accuracy producing complex morphological features, so as to meet the needs of repairing long segment nerve defects.

## Biomaterials for Additive Manufacturing of Nerve Guidance Conduits

Compared with traditional manufacturing methods (e.g., dip coating, electrospinning, molding), 3D printing has the advantages of being highly cost-effective and having high production efficiency. Several reviews on biomaterials for NGCs have been published (Zhang et al., 2020a), so we will focus on the biomaterials used in additive manufacturing of NGCs in this section.

The advantage of natural materials is their good biocompatibility, but their mechanical properties are poor and purification is difficult. Synthetic polymers-based NGCs can be relatively easy to prepare and achieve good mechanical properties, but their biocompatibility is not as good as natural polymers.

### Natural Polymers

Natural polymer materials are characterized by suitable cellular histocompatibility and excellent degradation performance (Deumens et al., 2010; Zhang et al., 2020b). Therefore, they have been applied widely in tissue engineering, including regeneration of peripheral nerves. However, they have weak mechanical properties, and they carry the risk of antigenicity and disease transmission. Several natural materials, including collagen (Weng et al., 2012), hyaluronic acid (HA) (Suri et al., 2011), alginate (Lee and Mooney, 2012; Johnson et al., 2015), gelatin (Hu et al., 2016) and silk fibroin (SF) (Kim et al., 2018), have been used in additive manufacturing of NGCs.

Collagen types I, II, and III are critical components of peripheral nerves (Georgiou et al., 2015; Bozkurt et al., 2016). Collagen can simulate the structure and function of the extracellular matrix. Also, the promotion of axonal regeneration and myelination by gelatinized NGCs of various types of collagen has been demonstrated *in vitro* and *in vivo*. Several US Food and Drug Administration-approved collagen products are on the market, but they are suitable only for patients with nerve defects < 3 cm (Kehoe et al., 2012; Sarker et al., 2018).

Hyaluronic acid is also one of the essential components of the extracellular matrix, and its immunogenicity is low. The porous structure of HA scaffolds enables them to be used as suitable carriers for drugs and bioactive substances (Ikeda et al., 2003). HA enhances cell adhesion by binding adhesion molecules or peptides (Suri et al., 2011).

As a type of denatured collagen, gelatin is used widely in peripheral-nerve scaffolds. Several studies have shown that gelatin nerve ducts have good cell compatibility, excellent degradability, and promote axon regeneration (Hu et al., 2016). The methacryloyl gelatin (GelMA) obtained by functionalizing gelatin with methacryloyl substituent groups is often used as a material for 3D printing of NGCs. The methacryloyl substituent groups give GelMA photocrosslinking and polymerization functions, and the mechanical properties are improved (Hu et al., 2016; Zhu et al., 2018).

Silk fibroin comes from the fibrin of the silkworm, and many SF-based peripheral NGCs have been fabricated. SF can be used to produce films, gels, and sponges. Among them, Kim et al. used methacrylate-grouped SF to produce degradation properties using its photoactivation and free radical polymerization properties Adjustable hydrogel, and prepared into SF-biological ink for bioprinting (Kim et al., 2018).

### Synthetic Polymers

The advantages of synthetic materials are that they are convenient to produce, and their mechanical properties can be adjusted. However, compared with natural biological materials, their biocompatibility is poor. Several synthetic materials, such as PCL (Singh et al., 2018), poly(lactic acid-co-glycolic acid) (PLGA) (Radulescu et al., 2007), poly(ethylene glycol) (PEG) (Christopher et al., 2015; Evangelista et al., 2015), poly(glycerol sebacate) (PGS) (Dharaminder et al., 2018), polypyrrole (PPy) (Weng et al., 2012) and carbon nanotubes (CNTs) (Lee et al., 2018a) have been used alone or in combination with natural polymers for 3D-printed NGCs.

The aliphatic polyester PCL is used widely as a raw material for NGCs due to its excellent mechanical properties (Kim and Kim, 2007; Schnell et al., 2007; Chang et al., 2018; Huang et al., 2018). However, the degradation performance of PCL is poor. Compared with PCL, PLGA has an adjustable degradation performance and mechanical properties. The very permeable PLGA/Pluronic F127 NGC and chitosan/PLGA compound NGCs have been tested *in vivo* to promote motor-function recovery and axon regeneration (Oh et al., 2008; Xue et al., 2012).

Although PEG has excellent biocompatibility, its effect on nerve regeneration is not clear. However, during preparation of the NGC, the photocrosslinkable PEG and PEGDA can serve as cell carriers, and ∼87% of the cells embedded have good cellular activity (Arcaute et al., 2006). Hence, PEGDA has been used widely in the additive fabrication of PEG-based NGCs.

PGS is a photocurable and absorbable material that can be used as a 3D-printed material for NGCs (Dharaminder et al., 2018). PGS-based NGCs can provide mechanical properties close to those of peripheral nerves and greater flexibility. Compared with PLGA, flat-sheet PGS NGCs can better promote the adhesion and proliferation of Schwann cells and reduce the inflammatory response, but they lack the 3D structure of the extracellular matrix. Photocurable, functionalized PGS-based NGCs can have both excellent mechanical properties and individual customization requirements (Dharaminder et al., 2018).

As the latest generation of NGC materials, conductive materials mainly comprise conductive polymers and CNTs (Weng et al., 2012; Lee et al., 2018a). They can maintain the integrity of the electrical signal of the nerve pathway and further synergize electrical stimulation. However, they are difficult to process, insoluble, and have poor degradability, so often they are combined with natural polymers. As mentioned earlier, hybrid composite additive manufacturing NGCs have also been used, such as chitosan/PLGA NGC. Hybrid composite nerve conduits Because hybrid composite nerve conduits can combine the advantages of both natural and synthetic polymers, so they are a promising type of material combination in the future.

## Methods for Additive Manufacturing of Nerve Guidance Conduits

**SCHEME 1:**
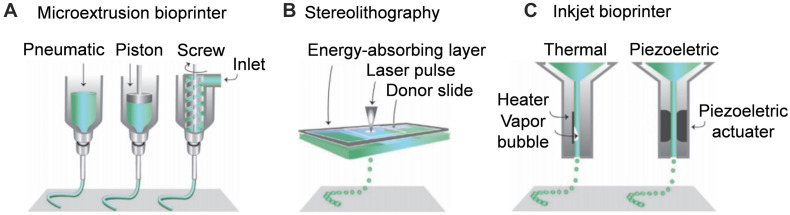
Schematic diagram of additive manufacturing techniques for nerve guide conduit. **(A)** Microextrusion bioprinter. **(B)** Stereolithography. **(C)** Inkjet bioprinter. Reproduced with permission from Malda et al. (2013).

3D printing is one of additive manufacturing technologies, which mainly refers to the method of obtaining 3D samples designed through continuous deposition of materials on the basis of computer assistance control. Continuous ink-jet printing has been around for nearly 70 years, and fused deposition modeling and stereolithography (SLA) were new technologies that emerged in the 1990. The three main methods of additive manufacturing of NGCs are extrusion-based printing, SLA, and inkjet bioprinting (Murphy and Atala, 2014; Lee et al., 2018a). Each method has its advantages and disadvantages. In this section, we introduce the principles of various additive manufacturing methods and their applications in NGC preparation (Scheme 1).

### Extrusion-Based Printing

**FIGURE 1:**
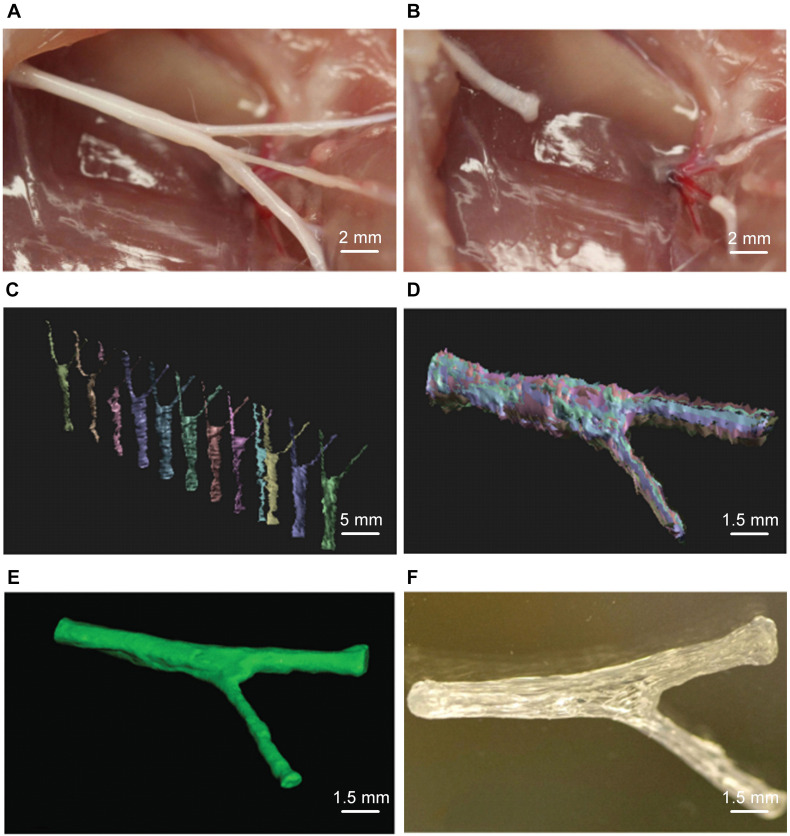
Microextrusion printing rat complex structure sciatic nerve conduit. **(A)** Photographs of sciatic nerve including both of sensory and motor nerve branches and **(B)** corresponding sciatic nerve defect. **(C)** Scans for 3D reconstruction from various perspectives by structured light scanning system. **(D)** The process of three-dimensional reconstruction of data. **(E)** Image after 3D reconstruction of the scan data of the sciatic nerve. **(F)** Image of the microextrusion printed sciatic nerve conduit. Reproduced with permission from Johnson et al. (2015).

Microextrusion printing can achieve one-step printing of NGCs containing various composite materials that are difficult to obtain by conventional manufacturing methods. Extrusion-based printing can obtain better mechanical properties and more complicated NGC structures than traditional production methods. Based on 3D imaging, Johnson et al. fabricated a sciatic nerve conduit with the bifurcation structure of sensory and motor branches by microextrusion printing (Figure 1). The disadvantage of extrusion 3D printing is that the efficiency and accuracy of printing are low due to the limitations of the nozzle, which is prone to blockage (Zhu et al., 2016). The printing materials are PCL, PLGA, alginate, calcium chloride, and GelMA. The delivery of NGF in the sensory branch and GDNF in the motor branch has been realized (Johnson et al., 2015).

Fused deposition modeling (FDM) is a printing technology. A temperature-sensitive polymer is heated to a molten state and then deposited on a solid medium through a printing nozzle (Qian et al., 2019). The advantage of this kind of technology is its high precision. The printed scaffold has high hardness, few impurities, and can eliminate organic solvents that are potentially toxic to cells. However, the polymer must be heated to a high temperature before printing, so the effect of high temperature on material properties must be considered.

Cui et al. (2009) fabricated double-layer PU/Col NGC by a modified FDM method. They used double needles, relatively low temperatures combined with separation and freezing to prepare the outer layer containing a macroporous (15–25 mm) structure of the inner layer of the oriented fiber PU/Col NGC. This NGC had excellent mechanical properties and biocompatibility, and the extrusion temperature was low, so growth factors and bioactive substances could be introduced.

**FIGURE 2:**
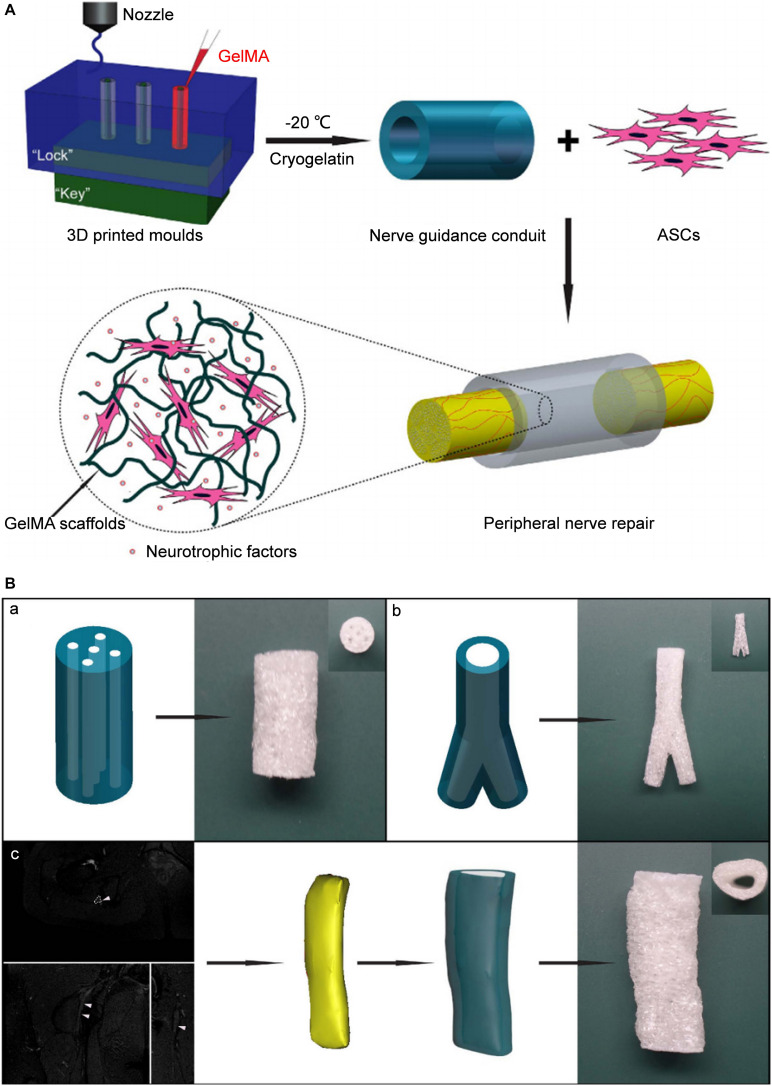
Schematic diagram of tissue engineered NGCs by indirect bioprinting, and computer models and photographs of complex structured NGCs. **(A)** Schematic diagram of tissue engineered nerve conduits. **(B)** Computer models and photographs of **(a)** 4-channel, **(b)** bifurcating. **(c)** MRI scan of the human sciatic nerve and the photograph of the corresponding nerve conduit. Reproduced with permission from Hu et al. (2016).

The indirect biological-printing method of microextrusion has also been used to produce NGCs. Hu et al. (2016) employed magnetic resonance imaging to obtain the data of the human sciatic nerve, and then used the indirect printing method to make a “personalized” GelMA NGC of the human sciatic nerve (Figure 2). By introducing GelMA hydrogel into the molds, they created a neural tube of a precise shape corresponding to the molds. When adipose-derived stem cells were introduced, the NGS could promote their adhesion and proliferation. *In vivo* experiments using a model of a 10-mm defect in the sciatic nerve showed that the nerve-repair effect was no different to that from an autograft group.

### Stereolithography

In-depth research into SLA (also known as photosensitive liquid phase solidification) has been done. SLA uses a laser light source to cure resins to produce complex 3D products. The main raw material added to the printing paste is a liquid resin. This photosensitive resin, after ultraviolet light (UV)-wavelength laser irradiation, causes the printing slurry to polymerize, and a single cured product is obtained. Then, the work platform is filled with a new printing slurry to continue the curing reaction of the next layer. This process is repeated layer-by-layer to obtain 3D solid parts. Finally, the latter are placed under UV light or a sintering furnace for molding.

The advantages of this technology are that: (i) macroscopic devices can be made under the control of a computer-aided design (CAD) or computer-aided manufacture; (ii) the accuracy and efficiency of production are higher than those of extrusion-printed methods. A high degree of automation means that the product is more controllable because it solidifies the liquid directly (Melchels et al., 2012). However, this method is suitable only for materials with high photosensitivity, and photoinitiators must be added, so tests of cytotoxicity and biocompatibility *in vitro* must be passed before use.

“Generalized SLA” comprises two types. One type is laser-assisted programmed SLA, which is characterized by projecting the image to be printed onto the polymer to increase the layer printing directly. The other type uses laser beams for point-by-point printing (Zhu et al., 2016).

**FIGURE 3:**
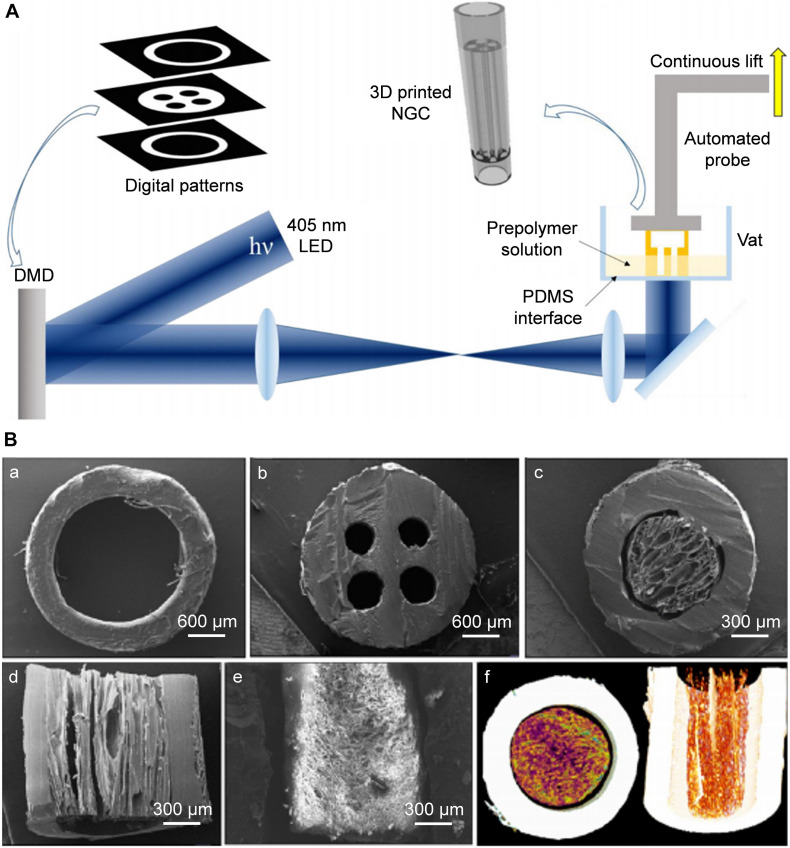
Rapid continuous 3D printing. **(A)** Schematic diagram of the rapid continuous 3D printing (Zhu et al., 2018). **(B)** SEM images of complex structured NGCs transverse sections of hollow **(a)**, 4-multichannel **(b)**, aligned cryomatrix-filled NGC **(c,d)**, random cryomatrix-filled **(e)**, Micro-CT image of aligned cryomatrix-filled NGC **(f)** (Singh et al., 2018). **(A)** Reproduced with permission from Zhu et al. (2018) and **(B)** Reproduced with permission from Singh et al. (2018).

Digital micromirror devices (DMDs) are employed widely in SLA to improve the printing efficiency. Zhu et al. (2018) used SLA to fabricate a human-facial-nerve conduit with bifurcated structures. After collecting the data of the NGCs to be printed by computed tomography or nuclear magnetic resonance spectroscopy, a CAD model was established. Using a 405-nm laser, light was transmitted into the GelMA and PEGDA prepolymer solution through a DMD to achieve selective light-curing according to CAD-model data (Figure 3A). This CAD design had the advantages of high efficiency, continuous and rapid customized printing, and preparation of NGCs with a multi-lumen and bifurcated structure.

Singh et al. (2018) used SLA to produce a 3D-printed PCL resin with reactive methacrylate groups. The preparation parameters of the laser light source was 400–500 nm, the thickness of each layer was 25 μm, and a PCL NGC of length 1.9 cm containing four microchannels and an accuracy of 50 μm was fabricated (Figure 3B). This PCL NGC filled with a nerve growth factor-loaded aligned cytomatrix repaired the critical length defect of a rat sciatic nerve.

Ye et al. (2020) used digital light-processing 3D printing technology to prepare a four-lumen GelMA hydrogel NGC. *In vitro* experiments demonstrated that the NGC promoted the proliferation of PC-12 cells, the directional migration along the long axis of the NGC, and promoted the directional differentiation of neurons of neural crest stem cells. These observations showed great potential for application in peripheral-nerve repair, but the study did not compare multi-channel with single-channel NGCs (Ye). PEG resin has also been employed in the fabrication of NGCs using SLA (Christopher et al., 2015; Evangelista et al., 2015). Evangelista et al. (2015) compared single-lumen NGCs with multi-lumen NGCs fabricated by SLA. They found that the effect of single-lumen conduit on sciatic-nerve regeneration was better than that of multi-lumen PEG NGCs (Evangelista et al., 2015). Dharaminder et al. (2018) prepared photocurable functionalized PGS through methacrylation of hydroxyl end-groups. A 405-nm laser was used to photocure PGSm, and the printing speed was 0.3 mm/s. After printing, methanol solution was used to remove uncured PGSm, and both ends of the NGC were laser-cut to obtain a complete 1.5 cm-long PGSm NGC. The mechanical-performance test results showed that its compressive Young’s modulus was >3 MPa and could fully resist the tension of the suture. *In vitro* experiments with S100-β immunofluorescence showed that the PGS-coated material could maintain the morphology of Schwann cells. *In vivo* tests demonstrated that the PGSm NGC promoted the regeneration of the common fibular nerve and reduced neuralgia (Dharaminder et al., 2018).

Conductive materials can also be applied in 3D-printed NGCs using SLA. Lee et al. fabricated 3D-printed MWCNTs-loaded PEGDA scaffolds using SLA, and *in vitro* experiments showed that synergistic electrical stimulation could promote the growth of neurites of neural stem cells (Lee et al., 2018b). Heo et al. (2019) used poly(3,4-ethylene dioxythiophene):polystyrene sulfonate (PEDOT:PSS) to further increase the conductivity of nerve scaffolds. This PEDOT:PSS/PEGDA scaffold could enhance the neuronal differentiation of dorsal root ganglion cells. However, few *in vivo* studies on this type of NGC have been done, and its effectiveness merits further study.

### Inkjet Bioprinting

Inkjet bioprinting is a commonly used additive technology, but it is not used widely used in NGCs (Dixon et al., 2018). Inkjet-printing technology can use polymers as raw materials, and accurately control the speed of polymer deposition droplets on a 3D coordinate axis through non-contact additive manufacturing technology. The power of the droplet’s advancement takes two forms: (i) the air pressure generated by heat; (ii) the pulse pressure generated by piezoelectric or ultrasonic devices. Then, the droplet is delivered to the substrate that supports or becomes part of the final product (Okamoto et al., 2000).

The earliest batch of inkjet-bioprinting equipment was modified from 2D ink-based printers. That is, the original ink was replaced with the biological material that needs to be printed, and the paper was changed to the corresponding Z-axis that can be moved up and down (Xu et al., 2008). Radulescu et al. (2007) demonstrated the compatibility of human embryonic kidney cells and promotion of nerve grow the factor, and adjusted the parameters of the inkjet printer to prepare additively manufactured cylindrical PLGA NGCs. By genetic modification of human embryonic kidney cells, the NGC could be used as an effective carrier for growth factors (Radulescu et al., 2007).

**FIGURE 4:**
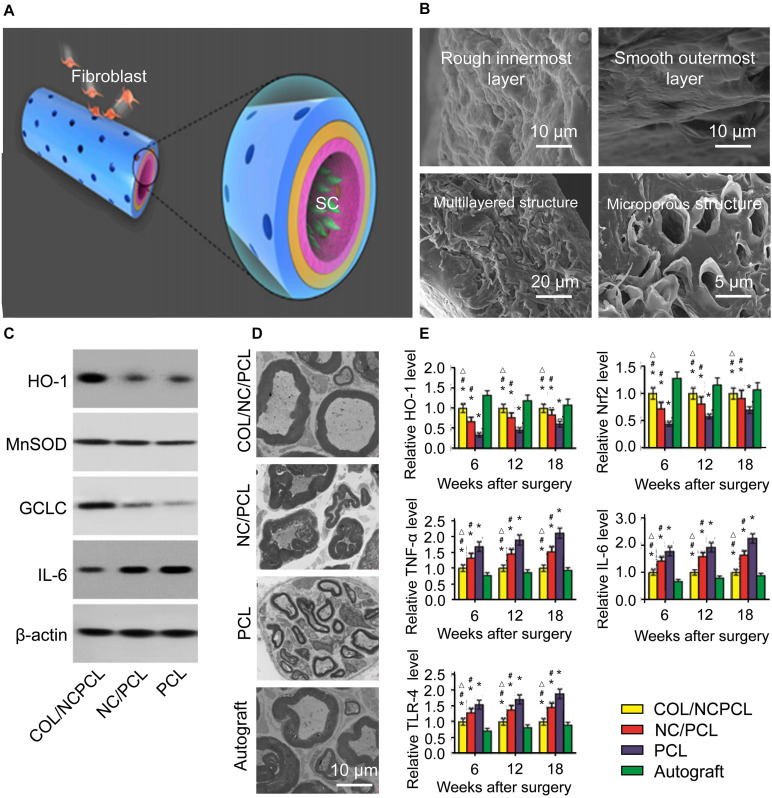
Schematic diagram of the 3D fabrication of the Col/NC/PCL NGCs, transmission electron micrograph of a sciatic nerve after surgery and the antioxidant properties of Col/NC/PCL NGCs (Qian et al., 2019). **(A)** The Col/NC/PCL NGC was printed in three layers from the outside to the inside, the Col layer, PCL layer, and NC/PCL layer. The inner layer was suitable for SC adhesion, and the outer layer could prevent fibroblasts from entering the conduit. **(B)** SEM of the Col/NC/PCL NGCs; **(C)** Western blot results of in vitro antioxidant and anti-inflammatory indicators; **(D)** Transmission electron micrograph of the sciatic nerve 18 weeks after surgery; **(E)** Reverse transcription-polymerase chain reaction (RT-PCR) results and the HO-1 and nuclear factor-like 2 (Nrf2) levels Reproduced with permission from Qian et al. (2019).

The performance of inkjet printing technology has facilitated introduction of functionally active substances. Qian et al. (2018) used inkjet bioprinting to prepare functional collagen/nanoceria/PCL NGCs to investigate the characteristics of inflammation and oxidative stress after nerve defects (Figure 4). They used a rotating roller with a structure of microneedles (simulating the pores of NGCs) to spray polymers on abrasive tools to prepare NGCs. *In vivo* and *in vitro* experiments demonstrated that the NGC had good local anti-oxidative stress function (Qian et al., 2019). Yuan and coworkers fabricated polydopamine- and arginyl glycyl aspartic acid-coated grapheme-loaded PCL nerve scaffolds by a similar type of inkjet bioprinting, and the NGC was used to repair a 15-mm sciatic-nerve defect in rats. The repair effect was no different from that of an autologous-nerve-graft group 18 months after the procedure, and study of the mechanism of nerve regeneration revealed promotion of axon- and myelin-related protein expression (Qian et al., 2018).

With the advancement of inkjet-dispensing technology, increasing numbers of bioactive materials have been used in inkjet printing to make tissue-engineered devices (Silva et al., 2007). Nerve scaffolds made of conductive materials can also be prepared using inkjet printing. Wallace and colleagues developed PPy/collagen platforms by inkjet printing (Weng et al., 2012). The electrical conductivity of these scaffolds was >1 S/cm, and bio-printing with micron precision was realized. *In vitro* tests demonstrated that the scaffold had good compatibility with PC-12 cells, and could promote the directional alignment and elongation of synapses upon synergistic electrical stimulation.

## Summary and Future Perspectives

Compared with the traditional manufacturing method, the nerve conduit manufactured by 3D printing has the advantages of low price, high efficiency, and easy preparation, and can be used as a growth factor or a carrier of bioactive substances. Further efforts will be directed toward the fabrication of NGCs with nano-precision and with growth factors or with growth factors gradient, as well as the development of new additive materials. The 3D bioprinting nerve conduit containing cells and growth factors, which can be used to better simulate the *in vivo* peripheral nerve micro-environment, is expected to repair peripheral nerve defects of limited length and will be the research direction of additive manufacturing of NGCs in the future.

## Author Contributions

SS wrote the manuscript. SS, XW, TW, QY, ZH, and ZZ revised the manuscript. ZZ and RL designed this work and revised the manuscript. All authors contributed to the article and approved the submitted version.

## Conflict of Interest

The authors declare that the research was conducted in the absence of any commercial or financial relationships that could be construed as a potential conflict of interest.
